# The SOX9-MMS22L Axis Promotes Oxaliplatin Resistance in Colorectal Cancer

**DOI:** 10.3389/fmolb.2021.646542

**Published:** 2021-05-27

**Authors:** Yiqiang Liu, Hong Wu, Tao Luo, Qiyu Luo, Ziyu Meng, Ying Shi, Feifei Li, Mingxin Liu, Xinhao Peng, Junjie Liu, Chuan Xu, Weizhong Tang

**Affiliations:** ^1^Department of Experimental Research, The Affiliated Tumor Hospital of Guangxi Medical University, Nanning, China; ^2^Integrative Cancer Center and Cancer Clinical Research Center, Sichuan Cancer Hospital and Institute, Sichuan Cancer Center, School of Medicine, University of Electronic Science and Technology of China, Chengdu, China

**Keywords:** colorectal cancer, SOX9, MMS22L, DNA damage repair, oxaliplatin resistance

## Abstract

**Background:**

Colorectal cancer (CRC) is estimated to be one of the most common cancers and the leading cause of cancer-related death worldwide. SOX9 is commonly overexpressed in CRC and participates in drug resistance. In addition, DNA damage repair confers resistance to anticancer drugs. However, the correlation between DNA damage repair and high SOX9 expression is still unclear. In this study, we aimed to investigate the function and the specific underlying mechanism of the SOX9-dependent DNA damage repair pathway in CRC.

**Methods:**

The expression levels of SOX9 and MMS22L in CRC were examined by immunohistochemistry (IHC) and TCGA analysis. RNA sequencing was conducted in RKO SOX9-deficient cells and RKO shControl cells. Mechanistic studies were performed in CRC cells by modulating SOX9 and MMS22L expression, and we evaluated drug sensitivity and DNA damage repair signaling events. In addition, we investigated the effect of oxaliplatin in tumors with SOX9 overexpression and low expression of MMS22L *in vivo*.

**Results:**

Our study showed that SOX9 has a higher expression level in CRC tissues than in normal tissues and predicts poor prognosis in CRC patients. Overexpression and knockdown of SOX9 were associated with the efficacy of oxaliplatin. In addition, SOX9 activity was enriched in the DNA damage repair pathway *via* regulation of MMS22L expression and participation in DNA double-strand break repair. SOX9 was upregulated and formed a complex with MMS22L, which promoted the nuclear translocation of MMS22L upon oxaliplatin treatment. Moreover, the *x*enograft assay results showed that oxaliplatin abrogated tumor growth from cells with MMS22L downregulation in mice.

**Conclusions:**

In CRC, activation of the SOX9-MMS22L-dependent DNA damage pathway is a core pathway regulating oxaliplatin sensitivity. Targeting this pathway in oxaliplatin-resistant CRC cells is a promising therapeutic option.

## Introduction

Colorectal cancer (CRC) is one of the most common cancers and the leading cause of cancer-related death worldwide (Siegel et al., 2020). CRC patients are generally diagnosed at an advanced stage, and death is usually caused by recurrence and metastasis to other organs (D. Chen et al., 2020). Drug resistance is not only the main cause of treatment failure but also the main factor that limits the survival of CRC patients (Carrasco-Garcia et al., 2016).

SOX9 is a transcription factor that has been reported to be linked with stem cell maintenance and is commonly overexpressed in solid tumors (Carrasco-Garcia et al., 2016; Christin et al., 2020; Kaushik et al., 2021). High expression of SOX9 in CRC correlates with advanced tumor stage and worse overall survival in patients (Lü et al., 2008). The oncogenic role of SOX9 is related to several cell signaling pathways, such as the TGFβ/Smad, Notch and Wnt/β-catenin pathways (Li and Neaves, 2006; Abdul Khalek et al., 2010). Studies have confirmed that SOX9-overexpressing cancer cells are resistant to oxaliplatin (Anuja et al., 2019; Qian et al., 2019; Abad et al., 2020; Das et al., 2020). However, the underlying mechanism of SOX9 in resistance to oxaliplatin is still unclear.

Oxaliplatin is a commonly used drug in the treatment of CRC and can inhibit DNA replication and transcription by promoting the formation of intra- and interstrand DNA breaks (Hochster and Sargent, 2016). Studies have shown that oxaliplatin adducts are less sensitive to the activity of DNA excision repair enzymes than native DNA and are equally damaging in DNA mismatch repair-proficient and DNA mismatch repair-deficient cells *in vitro* (Rothenberg, 2000). Understanding the molecular events controlling drug resistance is of paramount importance for cancer therapy. MMS22L is required for the repair of stalled/collapsed replication forks (O’Donnell et al., 2010). Studies have shown that human cells expressing low levels of MMS22L are sensitive to drugs that can induce replication fork reversal or Double-strand breaks (DSBs) at replication forks, such as camptothecin (CPT) (Duro et al., 2010; O’Connell et al., 2010). However, the regulatory role of MMS22L in SOX9-mediated chemotherapeutic resistance in CRC remains unclear. Therefore, identifying the role of the SOX9-DNA damage repair protein axis in anticancer drug resistance and aiming to elucidate its relevance to signaling pathway regulation may lead to the identification of new therapeutic targets.

## Materials and Methods

### Cell Lines and Chemical Reagents

The human colon cancer cell lines HCT116, HT-29, DLD1, RKO, and SW480 and the human normal colorectal mucosal cell line FHC were purchased from the American Type Culture Collection (ATCC) and cultured in Dulbecco’s modified Eagle’s medium (DMEM, CA, United States) supplemented with 10% fetal bovine serum (FBS, Jiangsu, China) and 100 U/ml penicillin-streptomycin. The culture conditions were a cell incubator with a temperature of 37°C and 5% carbon dioxide. Oxaliplatin was purchased from Selleck Chemical (Selleck, S1224, TX, United States).

### Patients and Tissue Specimen

CRC tumor and adjacent normal tissue slices were obtained from the Department of Pathology, Affiliated Tumor Hospital of Guangxi Medical University, Nanning, China, with approval from the Institutional Ethics Committee. All patients signed the informed consent form, and clinical data are provided in Supplementary Table 1.

### Lentiviral Infection Procedures

The CMV-MCS-3FLAG-SV40-Neomycin lentiviral Vector expressing full-length human SOX9 (NM_000346.4) and the U6-MCS-Ubiquitin-cherry-IRES-puromycin shSOX9 lentiviral Vector were purchased from GeneChem (Shanghai, China). The lentiviral packaging procedure was performed as described previously (Wu et al., 2020). RKO and SW480 cells (1 × 10^6^ cells/well) were infected with lentivirus. Stably transduced cells were selected and enriched with 4 μg/ml puromycin (Thermo Fisher Scientific, MA, United States). The sequences of the shRNA and the primers used to amplify the full-length sequence of human SOX9 are listed below:

SOX9-RNAi: forward: 5′-CCGGCTCCACCTTCACCTACAT GAACTCGAGTTCATGTAGGTGAAGGTGGAGTTTTTG-3′, reverse: 5′-GATCCAAAAACTCCACCTTCACCTACATGA ACTCGAGTTCATGTAGGTGAAGGTGGAG-3′.
SOX9-control: forward: 5′-CCGGTTCTCCGAACGTGTCA CGTCTCGAGTTCATGTAGGTGAAGGTGGAGTTTTTG-3′, reverse: 5′-GATCCAAAAATTCTCCGAACGTGTCACGTC TCGAGTTCATGTAGGTGAAGGTGGAG-3′.
MMS22L-RNAi: forward: 5′-CCGGCCAACTCTTCAAGGA AACTAACTCGAGTTAGTTTCCTTGAAGAGTTGGTTT TTG-3′, reverse: 5′-AATTCAAAAACCAACTCTTCAAGG AAACTAACTCGAGTTAGTTTCCTTGAAGAGTTGG-3′.
MMS22L-control: forward: 5′-CCGGTTCTCCGAACGTGT CACGTCTCGAGTTAGTTTCCTTGAAGAGTTGGTTTTTG-3′, reverse: 5′-AATTCAAAAATTCTCCGAACGTGTCACGTC TCGAGTTAGTTTCCTTGAAGAGTTGG-3′.
The primers used to amplify the full-length sequence of human SOX9 were as follows: forward: 5′-GAGGAAGTCG GTGAAGAACGG-3′, reverse: 5′-CTGAGCGGGGTTCATG TAGG-3′.

### RNA Sequencing and Bioinformatic Data Analysis

Total RNA was extracted from RKO shControl (shCtrl) and RKO shSOX9 cells. An RNA library was constructed using a TruSeq Stranded mRNA LT Sample Prep Kit (Illumina, CA, United States) and subjected to 125 bp/150 bp paired-end sequencing on an Illumina platform (HiSeq^TM^ 2500 or Illumina HiSeq^TM^ X Ten) by Haplox (Jiangxi, China). The differentially expressed genes (DEGs) were identified using Cuffdiff with q value < 0.05 and fold change >2 as the criteria. Gene set enrichment analysis (GSEA) was performed on the software.broadinstitute.org/gsea website. Volcano plots, pathway enrichment analysis visualizations and heatmaps were generated with the R program. All details were described in our previous work (Wu et al., 2020).

### Immunohistochemical Staining

Immunohistochemical staining was performed on tumor tissue slides as previously described (Wu et al., 2020). The slides were incubated with rabbit polyclonal anti-SOX9 (1:200, ABclonal, A19710, Hubei, China) and anti-MMS22L (1:200, Bioss, 17689R, Beijing, China) antibodies at 4°C overnight. SOX9 and MMS22L expression was evaluated by using a system considering the staining intensity (0 means negative; 1 means weak; 2 means moderate; and 3 means strong) and the percentage of positively stained cells (<5% = 0, 5% to <25% = 1, 25% to 50% = 2, > 50 to <75% = 3, ≥ 75% = 4). The final score was calculated by multiplying the extent score by the intensity score, and expression was accordingly classified as low or high with a score of four as the maximum value for low expression (Zou et al., 2019).

### Western Blotting

Total protein was obtained by the following steps. Cancer cells were lysed using radioimmunoprecipitation assay buffer (RIPA, Thermo Fisher Scientific, 89901, MA, United States):Phenylmethylsulfonyl Fluoride (PMSF, Beyotime, ST505, Shanghai, China) = 100:1 on ice for 30 min. Samples were centrifuged at 15,000 × *g* for 15 min; the supernatant was then collected, and 5 × loading buffer was added and heated at 95°C for 10 min. The nuclear fractions were separated using a Nuclear Extraction Kit (Beyotime, Shanghai, China) according to the manufacturer’s instructions, and protein was obtained as described above. The samples were separated by SDS-PAGE, and proteins were transferred to Polyvinylidene Difluoride (PVDF) membranes (Millipore, IPVH00010, MA, United States). After blocking in 5% milk-PBST for 1 h at room temperature, the membranes were incubated with primary antibodies at 4°C overnight and with secondary antibodies at room temperature for 1 h. Positive bands were visualized by enhanced chemiluminescence (ECL) (Millipore, WBKLS0100, MA, United States). Information on the primary antibodies is provided in Supplementary Table 2.

### Quantitative Real-Time Polymerase Chain Reaction

Total RNA was extracted from cancer cells using TRIzol Reagent (Invitrogen, United States), and quantitative real-time polymerase chain reaction (qRT-PCR) was performed using a SYBR Prime Script RT-PCR kit (TaKaRa, Osaka, Japan) in a CFX Connect Real-Time System (Bio-Rad, CA, United States). GAPDH was used as the internal control. The primer sequences are listed as follows:

SOX9: forward: 5′-AGGAAGCTCGCGGACCAGTAC-3′, reverse: 5′-GGTGGTCCTTCTTGTGCTGCAC-3′; GAPDH: forward: 5′-GTTCGTCATGGGTGTGAAC-3′, reverse: 5′-ATGGCATGGACTGTGGTCAT-3′; MMS22L: forward: 5′- ATGTGAGCGGGAATCTCTT-3′, reverse: 5′-GAAACACT TGGGGTTCGTC-3′.

### Immunofluorescence

Cells were seeded on coverslips (5 × 10^4^ cells per well) in a 24-well plate. 12 h after plating, cells were exposed to oxaliplatin at the indicated doses for 4 h. Then, cells were fixed with 4% paraformaldehyde and permeabilized with PBS containing 0.3% Triton X-100. After blocking with goat serum, cells were incubated with primary antibodies at 4°C overnight. Then, cells were incubated with fluorescent secondary antibodies (Molecular Probes, Eugene, OR, United States) (1:400) at room temperature for 30 min. Nuclei were stained with DAPI. A Nikon Eclipse Ti-S fluorescence microscope (Nikon, Tokyo, Japan) was used to acquire images. The paraffin sections required antigen repair prior to the above procedure.

### Cell Proliferation Assay

CRC cells were seeded in 96-well plates at a density of 2 × 10^3^ cells per well. Oxaliplatin was added to the culture medium at a concentration of 5 μM. Over the following 5 days, the CCK8 assay was performed daily. Cell Counting Kit-8 solution (Biosharp, BS350B, Anhui, China) was added to each well prior to incubation at 37°C for 2 h. Optical density was measured based on the absorbance at a wavelength of 450 nm.

### Flow Cytometric Apoptosis Analysis

CRC cells were treated with 5 μM oxaliplatin. According to the manufacturer’s instructions, the early apoptosis rate was analyzed by the Annexin-V staining method using a PE Annexin-V Apoptosis Detection Kit (Beyotime, C1065M, Shanghai, China) and a FITC Annexin-V Apoptosis Detection Kit (Beyotime, C1062M, Shanghai, China). We used FlowJo v.10 software for data analysis.

### Xenograft Assay

To evaluate the role of the SOX9-MMS22L axis *in vivo*, 4-week-old BALB/c nude mice (DOSSY, Chengdu, China) were subcutaneously inoculated with RKO (5 × 10^6^ cells/100 μl PBS) cells stably transfected with Vector+shCtrl, oeSOX9+shCtrl, Vector+shMMS22L, oeSOX9+shMMS22L plasmids. Mice were intraperitoneally injected with oxaliplatin (7.5 mg/kg) every 2 days for 10 days and sacrificed on Day 28 (Liu et al., 2020). The tumor volume was estimated every 4 days and calculated using the following formula: tumor volume = 0.5 × (length × width^2^). A portion of the xenograft tumors in the mice was taken for IHC staining. All animal experiments were performed in compliance with the guidelines of the Animal Ethics Committee of the Affiliated Tumor Hospital of Guangxi Medical University.

### Statistical Analysis

Statistical analysis was performed with GraphPad Prism 7.0 (CA, United States). One-way ANOVA or two-tailed unpaired *t*-tests were used to determine the level of significance. All data used in this study satisfied the assumptions of the statistical tests. All quantitative data are shown as the mean ± S.D. or mean ± S.E.M. values as indicated. *P* < 0.05 was considered statistically significant. All experiments were carried out at least three times.

## Results

### SOX9 Is Aberrantly Highly Expressed in Tumor Tissues and Predicts Poor Prognosis in CRC

**FIGURE 1:**
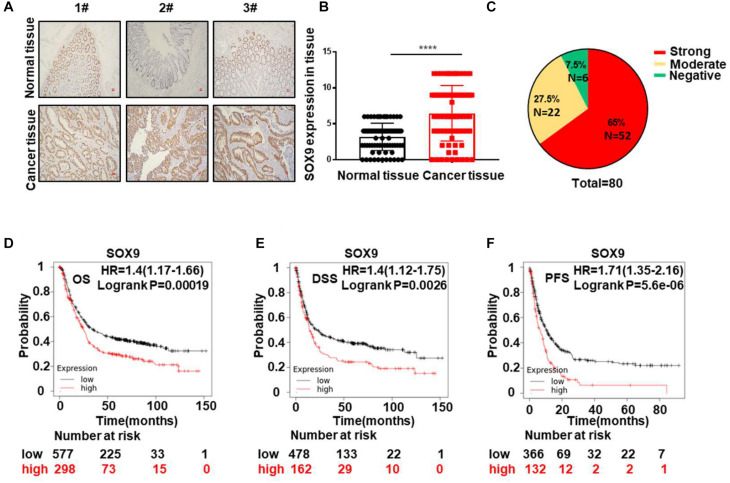
SOX9 is aberrantly highly expressed in tumor tissues and predicts poor prognosis in CRC. **(A)** Representative IHC staining image of the expression of SOX9 in three pairs of CRC tissues (T) and paired adjacent normal tissues (N). Scale bar = 50 μm. **(B)** Quantitative analysis of SOX9 expression in 80 CRC tumor tissues (T) and adjacent normal tissues (N). **(C)** Percentages of negative, moderate, and strong SOX9 expression in CRC tumor tissues (*n* = 80). **(D–F)** Kaplan–Meier analysis of the overall survival (OS) **(D)** and disease-specific survival (DSS) rates. **(E)** Progression-free survival (PFS) **(F)** in CRC patients with SOX9^low^ and SOX9^high^ tumors in the TCGA database. *P < 0.05, **P < 0.01, and ****P < 0.0001.

To verify the expression status of SOX9 in CRC patients, we examined SOX9 expression in 80 pairs of CRC tissues by IHC staining. Our results confirmed the high expression of SOX9 in tumor tissues compared to the adjacent non-tumor tissues (Figures 1A,B). In addition, 65% of CRC patients had strong SOX9 expression (Figure 1C). Similar results were observed in CRC cells (HCT116, HT-29, DLD1, SW480, and RKO) compared with human normal colorectal mucosal cells (FHC) (Supplementary Figure 4). Furthermore, SOX9^high^ CRC patients showed shorter overall survival, disease-specific survival and progression-free survival times than SOX9^low^ patients in the TCGA database (Figures 1D–F). Conclusively, these data indicate that SOX9 is aberrantly highly expressed in tumor tissues and predicts poor prognosis in CRC patients.

### SOX9 Increases Oxaliplatin Resistance and DNA Repair in CRC

**FIGURE 2:**
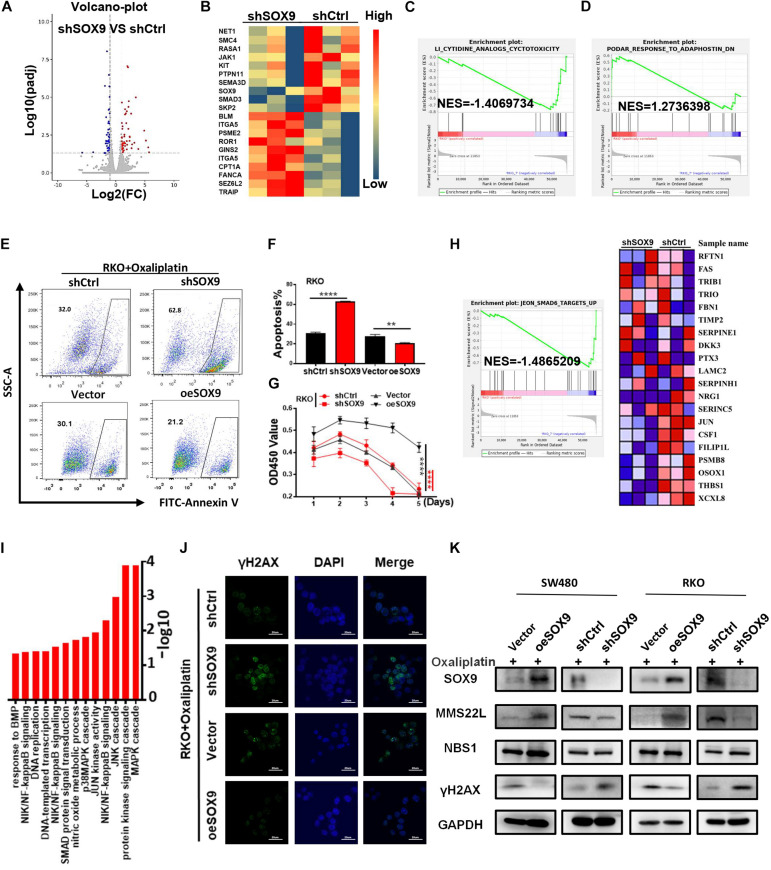
High expression of SOX9 promotes oxaliplatin resistance by inducing DNA repair in CRC cells. **(A)** Volcano plot showing the change in gene expression levels in shSOX9 RKO cells compared to control cells. **(B)** Heatmap showing the change in chemosensitivity and chemoresistance genes in shSOX9 RKO cells compared to control cells. **(C,D)** GSEA of drug sensitivity genes altered by SOX9 knockdown. **(E,F)** Representative images **(E)** and numbers **(F)** of early apoptotic shSOX9 and oeSOX9 CRC cells compared to control cells. **(G)** Cytotoxicity assay showing the proliferation of oeSOX9 and shSOX9 CRC cells treated with oxaliplatin compared to control cells. **(H)** GSEA of genes related to DNA damage altered by SOX9 knockdown in CRC cells. **(I)** Pathway enrichment analysis showing that the pathways related to DNA damage in shSOX9 CRC cells were extremely different from those in control cells. **(J)** Immunofluorescence showing representative images of γH2AX (green) in oeSOX9 and shSOX9 CRC cells treated with oxaliplatin compared to control cells. Nuclei were stained with DAPI (blue) (scale bar = 20 μm). **(K)** Western blotting was used to detect the expression of MMS22L, NBS1, and γH2AX in oeSOX9 and shSOX9 CRC cells treated with oxaliplatin compared to control cells. **p < 0.01; ***p < 0.001; and ****p < 0.0001.

To further explore the role of SOX9 in CRC cells, we established CRC cells with SOX9 knockdown and overexpression, termed shSOX9 and oeSOX9 cells, respectively (Supplementary Figures 1A–D). Next, we analyzed shSOX9 and shCtrl RKO cells by transcriptome sequencing, and the results showed that drug sensitivity-related genes were significantly enriched in CRC cells with low SOX9 expression (Figures 2A–D). We detected the early apoptosis rate and cytotoxicity of colorectal cells treated with oxaliplatin. The results showed that loss of SOX9 significantly increased the sensitivity of cells to oxaliplatin, while gain of SOX9 decreased the drug sensitivity of CRC cells (Figures 2E–G and Supplementary Figure 2). To further explore the underlying mechanism of SOX9 in promoting oxaliplatin resistance in CRC cells, we analyzed the transcriptome sequencing results by GSEA and pathway enrichment analysis. The results showed that there were correlations between SOX9 and the DNA damage response (Figures 2H,I). Immunofluorescence and western blot analyses showed that the expression of γH2AX in shSOX9 RKO cells was higher than that in shCtrl cells. However, the expression of γH2AX in oeSOX9 RKO cells was lower than that in Vector cells (Figure 2J). Next, western blotting was conducted to detect the expression of the classical molecules in the DNA damage repair pathway. We found that loss or gain of SOX9 up- or downregulated MMS22L expression, respectively, but had no effect on the other molecules (Figure 2K). In summary, our data suggested that SOX9 affected the sensitivity of CRC cells to oxaliplatin by participating in DNA damage repair.

### SOX9 Interacts With MMS22L to Promote DNA Repair in CRC Cells

**FIGURE 3:**
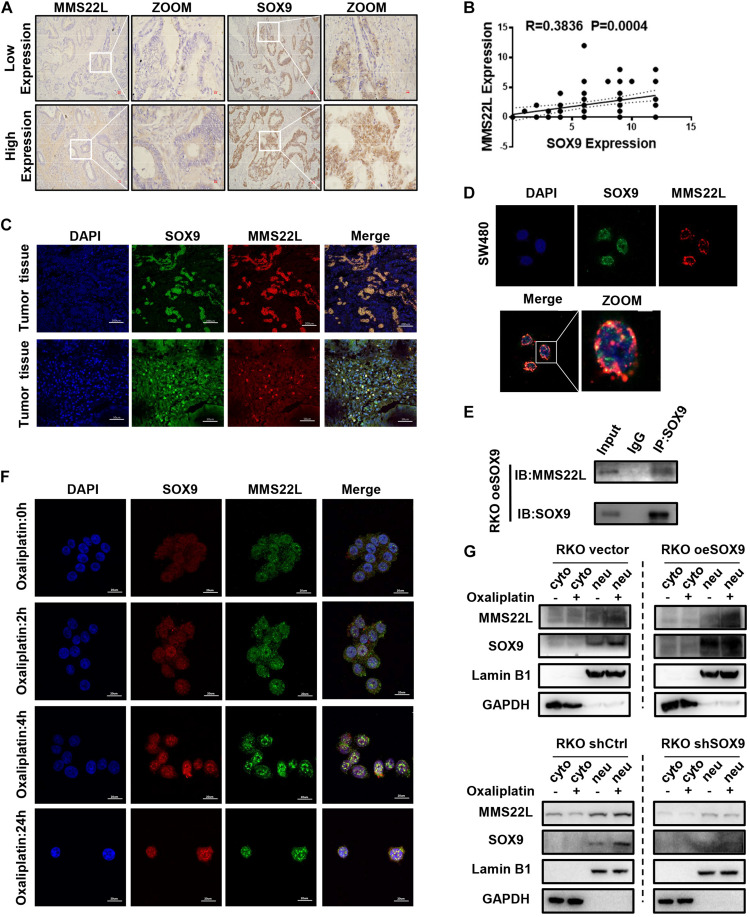
SOX9 enhances chemotherapeutic insensitivity in colorectal cancer by promoting MMS22L expression and nuclear translocation. **(A)** Representative images showing IHC staining of SOX9 and MMS22L in 2 CRC patients. Scale bar = 50 μm. **(B)** The correlation between SOX9 and MMS22L expression was analyzed in 80 CRC patients. **(C,D)** Representative immunofluorescence images showing SOX9 and MMS22L colocalization in CRC tissues and cell lines (scale bar = 20, 50, or 200 μm). **(E)** IP analysis of the interaction of MMS22L with SOX9 in SOX9-overexpressing RKO cells. **(F)** Immunofluorescence images showing the time-dependent variation in SOX9 and MMS22L colocalization in RKO cells treated with oxaliplatin. Scale bar = 20 μm. **(G)** Nuclear fractions were extracted from CRC cells, and the nuclear (neu) and cytoplasmic (without the nucleus; cyto) fractions were prepared. Immunoblot showing the protein levels of MMS22L and SOX9 expressed in Vector and oeSOX9 RKO cells and in shCtrl and shSOX9 RKO cells.

To further verify the relationship between SOX9 and MMS22L, we performed IHC staining of sections from 80 CRC patients. The results showed that the expression of SOX9 and MMS22L had a positive correlation (Figures 3A,B). Moreover, immunofluorescence costaining of these sections showed that SOX9 and MMS22L were colocalized in CRC tissues and cells (Figures 3C,D). Co-IP analysis of SOX9 and MMS22L in CRC cells confirmed this finding (Figure 3E). Furthermore, the SOX9- MMS22L interaction increased with the exposure time to oxaliplatin treatment (Figure 3F). Gain of SOX9 expression promoted the nuclear translocation of MMS22L upon oxaliplatin treatment (Figure 3G). Furthermore, the absence of SOX9 in RKO cells led to the opposite effects (Figure 3G). In summary, our data showed that SOX9 participated in DNA damage repair by promoting the expression and nuclear entry of MMS22L upon oxaliplatin treatment, which induced oxaliplatin resistance in CRC cells.

### The SOX9/MMS22L Axis Regulates Oxaliplatin Resistance in CRC

**FIGURE 4:**
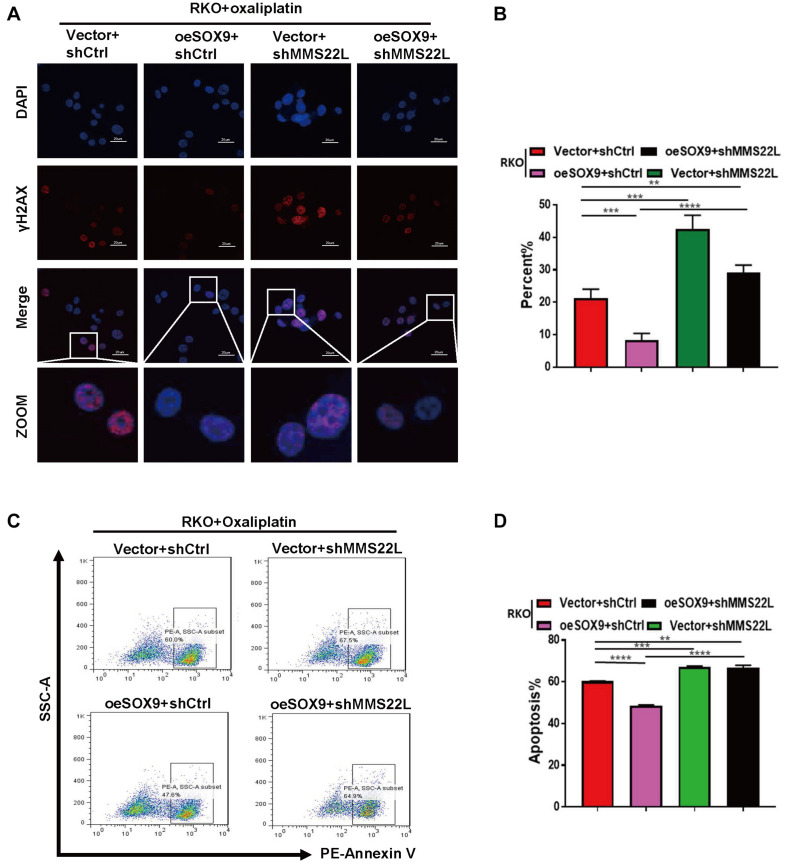
Loss of MMS22L enhances chemosensitivity in SOX9-overexpressing CRC cells. **(A,B)** Immunofluorescence images showing the typical appearance **(A)** and percentages **(B)** of γH2AX-positive cells in the indicated groups; nuclei were stained with DAPI (blue). Scale bar = 20 μm. **(C,D)** Flow cytometry results showing early apoptosis and the percentage of apoptotic cells among Vector+shCtrl, oeSOX9+shCtrl, Vector+shMMS22L, and oeSOX9+shMMS22L cells treated with oxaliplatin. **p < 0.01; ***p < 0.001; and ****p < 0.0001.

To explore the mechanism of oxaliplatin resistance in CRC cells with high SOX9 expression, we established shMMS22L and oeSOX9+shMMS22L RKO cells (Supplementary Figures 1E,F). Consistent with the previous results, immunofluorescence showed that overexpression of SOX9 augmented DNA repair under oxaliplatin treatment in CRC cells, but oeSOX9 RKO cells with loss of MMS22L showed less DNA repair activity than Vector RKO cells (Figures 4A,B). The results of early apoptosis detection by flow cytometry further demonstrated that MMS22L abolished the effects of SOX9 overexpression in cells treated with oxaliplatin (Figures 4C,D). In summary, our data indicated that SOX9 promoted oxaliplatin resistance in CRC cells through its interaction with MMS22L.

### The SOX9/MMS22L Axis Regulates Oxaliplatin Resistance *in vivo*

**FIGURE 5:**
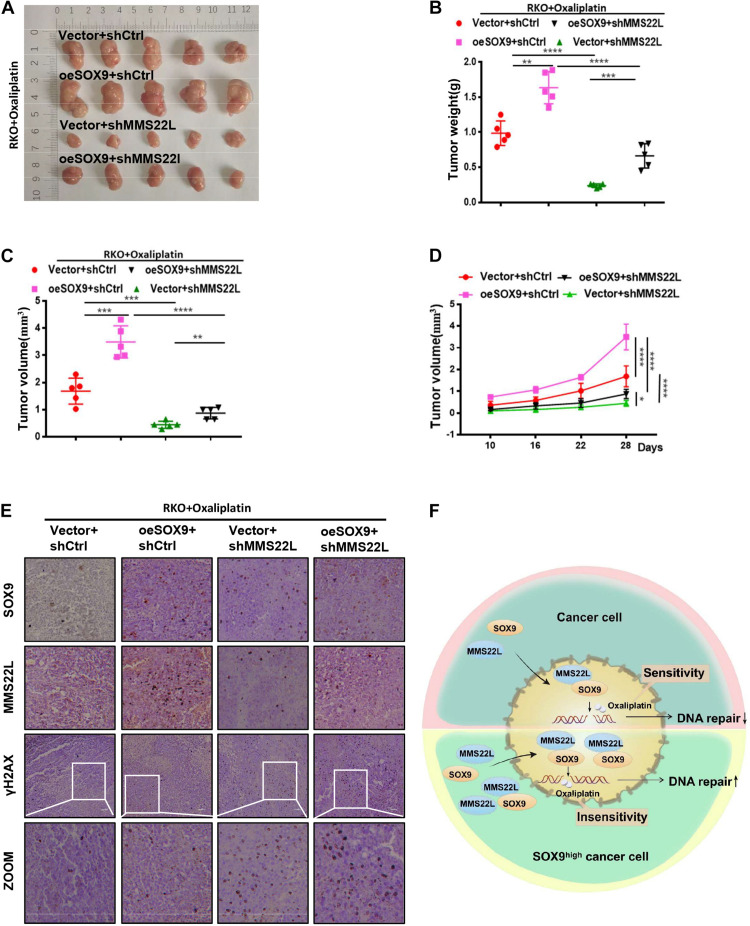
Loss of MMS22L enhances chemosensitivity in SOX9-overexpressing CRC cells *in vivo*. **(A–D)** Antitumor outcomes of RKO xenografts in male nude mice treated with oxaliplatin, including photographs of the tumors **(A)**, average tumor weights **(B)**, and tumor volumes **(C)**, at the end of the experimental period, along with the time-dependent variation curves of tumor volume **(D)**. **(E)** IHC staining images showing the expression of SOX9, MMS22L, and γH2AX in xenografts formed from Vector+shCtrl, oeSOX9+shCtrl, Vector+shMMS22L, and oeSOX9+shMMS22L cells. Scale bar = 50 μm. **(F)** Schematic diagram of the regulatory mechanism by which SOX9-mediated expression and nuclear translocation of MMS22L promotes DNA damage repair in CRC cells. **p < 0.01; ***p < 0.001; and ****p < 0.0001.

To further demonstrate the effect of SOX9 on CRC cells *in vivo*, we established a subcutaneous xenograft model in nude mice. Mice were implanted with Vector +shCtrl, oeSOX9+shCtrl, Vector+shMMS22L or oeSOX9+shMMS22L RKO cells and treated with oxaliplatin (75 mg/kg) (Figure 5A). After 28 days, the xenografts were harvested. The results showed that oxaliplatin had better therapeutic efficacy against oeSOX9+shMMS22L RKO tumors than oeSOX9+shCtrl tumors (Figures 5A–D). IHC staining showed that the expression of γH2AX in the oeSOX9+shCtrl group was lower than that in the oeSOX9+shMMS22L group (Figure 5E). Therefore, these results indicate that SOX9 upregulation increases oxaliplatin resistance in CRC cells *in vivo* and that knocking down MMS22L reverses this effect.

## Discussion

Cancer cells aquire drug resistance through different mechanisms (Chen and Shen, 2020; da Costa and Baiocchi, 2020; Qin et al., 2020; Zhu et al., 2020; Pastushenko et al., 2021), including enhancement of oxygen free radical scavenging, promotion of DNA damage repair, or inhibition of apoptotic signaling pathway activation (Lü et al., 2008; Bakkenist et al., 2018; Phi et al., 2018). *In vitro* studies suggest a role for SOX9 in regulating cell differentiation in the intestinal epithelium (Mori-Akiyama et al., 2007; Aguilar-Medina et al., 2019). A transcription factor related to stem cell maintenance, SOX9 is highly expressed in CRC and indicates poor prognosis (Aguilar-Medina et al., 2019). In human specimens, we confirmed that the expression of SOX9 in CRC was significantly upregulated compared to that in adjacent tissues and verified that SOX9 upregulation can promote the formation of CRC stem cells (Supplementary Figures 3A–C), similar to previous results. Recent studies on CRC have found that the overexpression of SOX9 in CRC is related to anticancer drug resistance, but the specific mechanism of drug resistance is not well studied. Considering the characteristics of cancer stem cells, we explored whether SOX9 participates in anticancer drug resistance by affecting DNA damage repair in cancer cells. Our results showed that SOX9 can affect the sensitivity of cancer cells to oxaliplatin by affecting the DNA damage repair. Overexpression of SOX9 significantly enhanced DNA damage repair, thus increasing the resistance of cancer cells to oxaliplatin. After knockdown of SOX9, this effect was significantly reversed. Moreover, we observed the expression of classical DNA damage repair-related molecules after knocking down or overexpressing SOX9 and found that SOX9 can affect MMS22L expression.

MMS22L is mainly localized in the nucleus and has been reported to be critical for protecting against DSBs formation in S-phase cells by dealing with replication fork stalling/collapse events (Piwko et al., 2011). Further research has shown that MMS22L participates in DNA repair by promoting the displacement of the single-stranded DNA binding protein RPA and assembly of RAD51 filaments at DNA damage sites in response to CPT (Piwko et al., 2016; Huang et al., 2018). It has been reported that SOX9 can usually influence related pathways by regulating the transcription of certain molecules. However, knockdown of SOX9 did not alter the mRNA level of MMS22L. In this study, we further confirmed an interaction between SOX9 and MMS22L by immunofluorescence and immunoprecipitation experiments. Furthermore, nucleocytoplasmic separation further confirmed that SOX9 increased the enrichment of MMS22L in the nucleus under treatment with oxaliplatin. Therefore, our results elucidated that SOX9 can participate in DNA damage repair by affecting the expression and nuclear translocation of MMS22L (Figure 5F).

In this manuscript, through overexpression or knockdown of SOX9 in CRC cells and a series of subsequent experiments, we elucidated that SOX9 can affect the sensitivity of CRC cells to oxaliplatin and preliminarily explored the mechanism of this phenomenon. However, there was a relative absence of SOX9 expression in drug-resistant CRC cells compared with wild-type cells. Therefore, we will screen oxaliplatin-resistant CRC cells in our next study and continue to perform more in-depth research. In conclusion, our study confirmed that SOX9, a transcription factor, is involved in the maintenance of stemness and the induction of drug resistance in CRC cells. Furthermore, we revealed a mechanism by which SOX9 regulates the expression and nuclear translocation of MMS22L and oxaliplatin resistance in CRC cells and provided a new focus for solving the problem of oxaliplatin resistance in CRC patients.

## Data Availability Statement

The data presented in the study are deposited in the SRA repository, accession number PRJNA707264.

## Ethics Statement

The animal study was reviewed and approved by the Ethics Committee of Affiliated Tumor Hospital of Guangxi Medical University. All authors have consented to publication of the results presented in this manuscript.

## Author Contributions

YL, HW, and TL performed the experiments. YL, HW, TL, QL, ZM, YS, FL, ML, XP, and JL provided technical support. CX, WT, XP, and JL provided critical comments. YL, HW, TL, CX, WT, XP, and JL analyzed the data, designed the experiments, and wrote the manuscript. All authors read and approved the final manuscript.

## Conflict of Interest

The authors declare that the research was conducted in the absence of any commercial or financial relationships that could be construed as a potential conflict of interest.
