# Supplementary figures and images for: The SOX9-MMS22L Axis Promotes Oxaliplatin Resistance in Colorectal Cancer
Source: Front Mol Biosci. 2021 May 27;8:646542. doi: 10.3389/fmolb.2021.646542 (PMC8191464; doi:10.3389/fmolb.2021.646542)

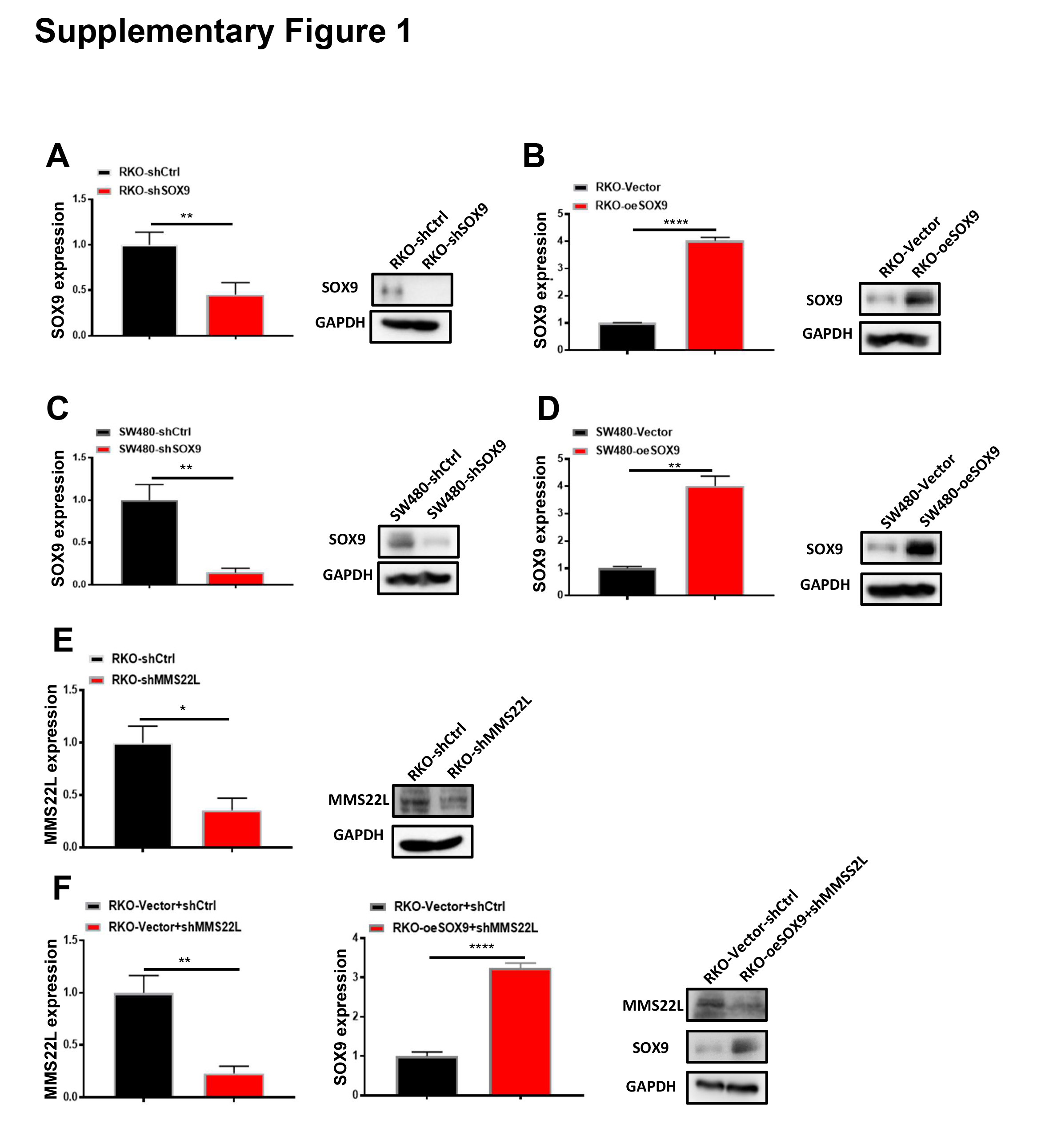

Supplement: Supplementary file 1 [file Image_1.jpg]

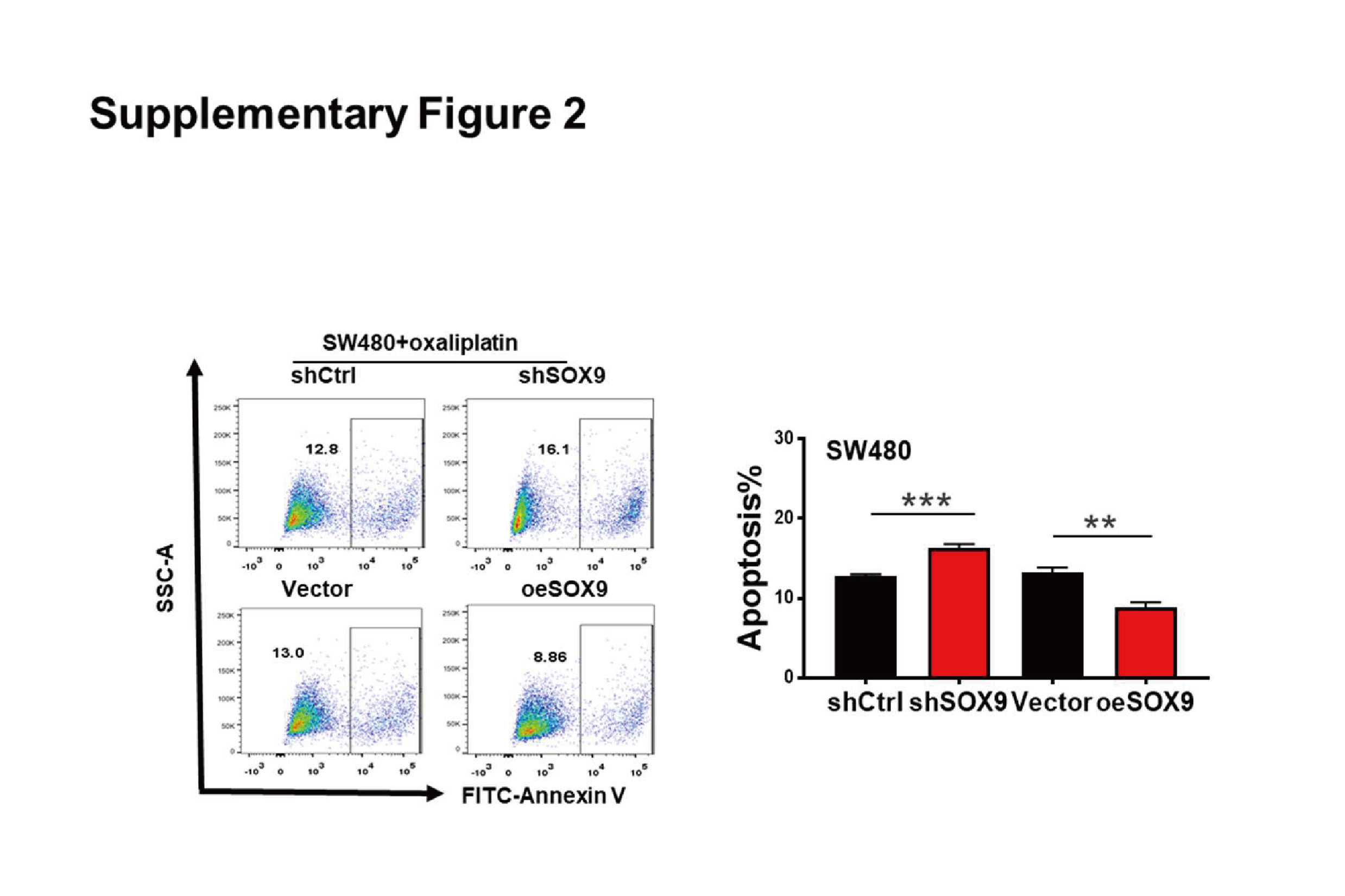

Supplement: Supplementary file 2 [file Image_2.JPEG]

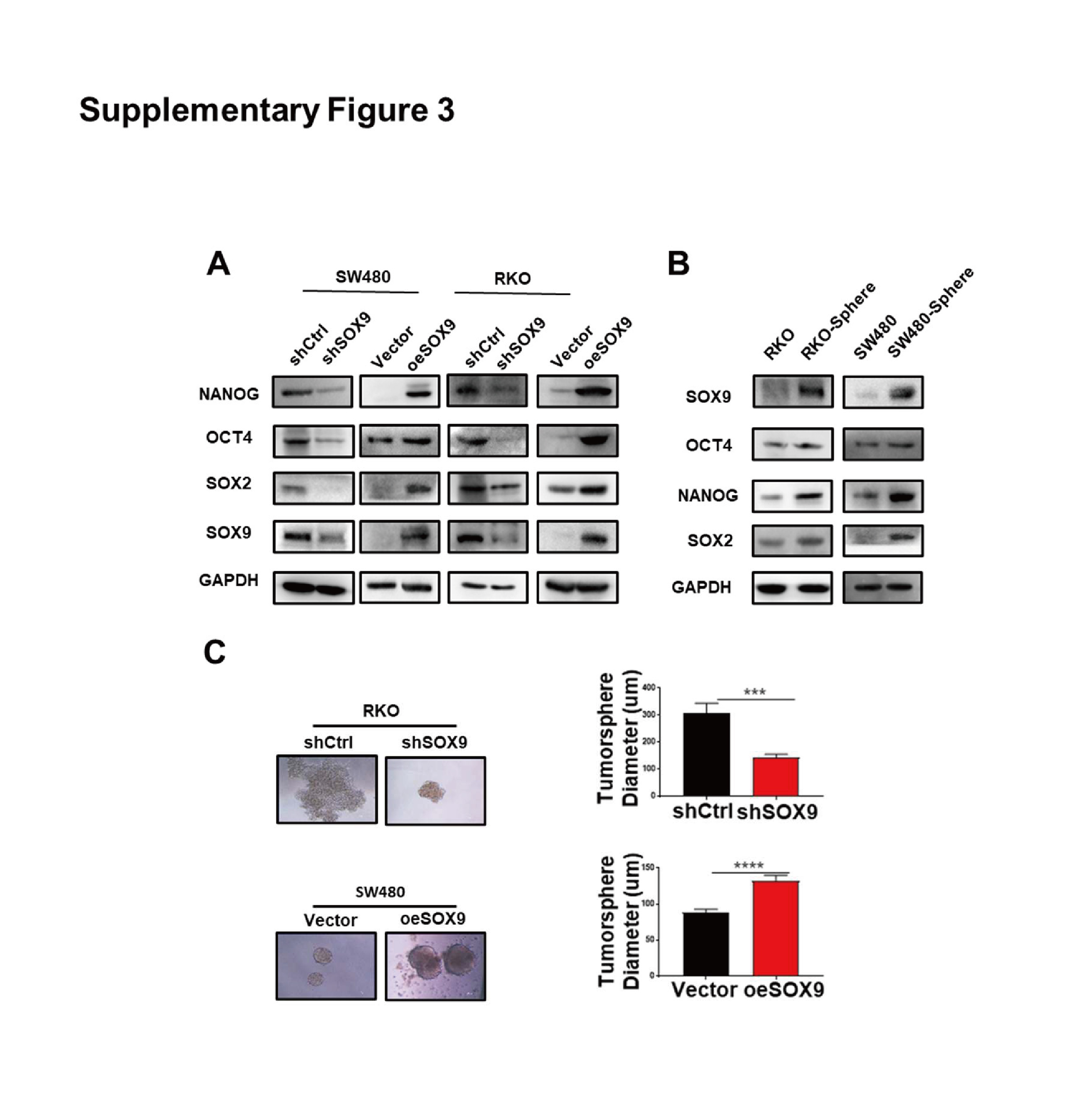

Supplement: Supplementary file 3 [file Image_3.JPEG]

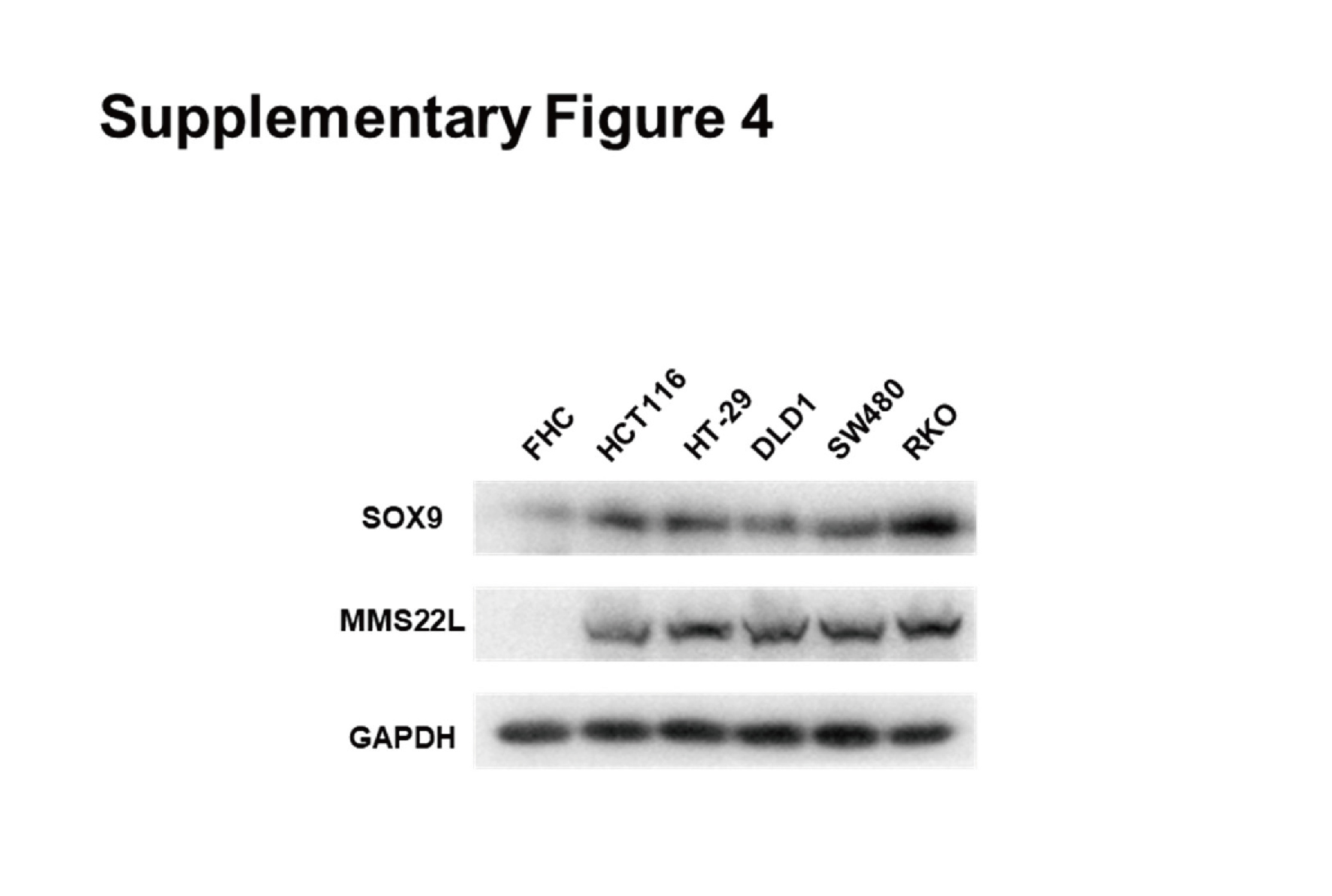

Supplement: Supplementary file 4 [file Image_4.JPEG]
