# Supplementary material for: The SOX9-MMS22L Axis Promotes Oxaliplatin Resistance in Colorectal Cancer
Source: Front Mol Biosci. 2021 May 27;8:646542. doi: 10.3389/fmolb.2021.646542 (PMC8191464; doi:10.3389/fmolb.2021.646542)
Supplement: Supplementary file 5 [file Data_Sheet_1.DOCX]

**FIGURE S1 |** The mRNA and protein levels of SOX9 and MMS22L in CRC cells. **(A-B)** Western blotting and qRT-PCR analysis of the abundances of SOX9 in up- and downregulated SOX9 groups compared to control groups in RKO cells. **(C-D)** Western blotting and qRT-PCR analysis of the abundances of SOX9 in up- and downregulated SOX9 groups compared to control groups in SW480 cells. **(E)** Western blotting and qRT-PCR analysis of the abundances of MMS22L in downregulated MMS22L groups compared to control groups in RKO cells. **(F)** Western blotting and qRT-PCR analysis of the abundances of SOX9 and MMS22L in oeSOX9+shMMS22L RKO cells compared with control groups.

**FIGURE S2 |** The representative images and numbers of early apoptosis in shSOX9 and oeSOX9 SW480 cells compared to control groups.

**FIGURE S3 |** SOX9 promotes the formation of colorectal cancer cell spheres**. (A)** Western blotting analysis of the abundances of NANOG, OCT4 and SOX2 in up- and downregulated SOX9 cells compared to control groups. **(B)** Western blotting analysis of SOX9 expression in the tumorsphere CRC cells. **(C)** The representative images and numbers of tumorsphere in oeSOX9 group and shSOX9 group compared to the control groups by tumorsphere formation assay, scale bar=50μm.

**FIGURE S4 |** Western blotting analysis of the abundances of SOX9 and MMS22L in CRC cells compared with normal colorectal mucosal cells.
