# Supplementary material for: The SOX9-MMS22L Axis Promotes Oxaliplatin Resistance in Colorectal Cancer
Source: Front Mol Biosci. 2021 May 27;8:646542. doi: 10.3389/fmolb.2021.646542 (PMC8191464; doi:10.3389/fmolb.2021.646542)
Supplement: Supplementary file 6 [file Table_1.DOCX]

**Supplementary Table 1. Clinicopathological features of CRC patients.**

| **Accession number** | **Gender** | **Age (years)** | **pT status** | **pN status** | **pM status** | **Stage** | **IHC Staining score** |
| --- | --- | --- | --- | --- | --- | --- | --- |
| 179076 | F | 67 | 3 | 2b | 0 | IIIC | 6 |
| 178992 | M | 62 | 2 | 0 | 0 | I | 9 |
| 179308 | F | 50 | 3 | 0 | 0 | IIA | 9 |
| 179327 | M | 47 | 3 | 1a | 0 | IIIB | 6 |
| 179185 | F | 49 | 3 | 0 | 0 | IIA | 4 |
| 179291 | M | 87 | 2 | 0 | 0 | I | 9 |
| 179386 | F | 70 | 3 | 2b | 0 | IIIC | 6 |
| 179285 | M | 53 | 3 | 0 | 0 | IIA | 12 |
| 179391 | F | 68 | 3 | 1a | 0 | IIIB | 9 |
| 177169 | M | 69 | 3 | 1a | 0 | IIIB | 9 |
| 177642 | F | 43 | 3 | 0 | 0 | IIA | 9 |
| 179455 | M | 51 | 3 | 1a | 0 | IIIB | 9 |
| 179611 | M | 67 | 3 | 1b | 1b | VIB | 9 |
| 179509 | F | 64 | 4a | 2a | 0 | IIIC | 9 |
| 179622 | M | 47 | 2 | 0 | 0 | I | 12 |
| 179404 | F | 75 | 3 | 1a | 0 | IIIB | 6 |
| 176887 | F | 57 | 4b | 0 | 0 | IIC | 6 |
| 177019 | M | 53 | 3 | 0 | 0 | IIA | 12 |
| 180022 | M | 56 | 3 | 0 | 0 | IIA | 12 |
| 179733 | F | 72 | 4b | 1a、1c | 1c | Ivc | 6 |
| 179892 | M | 56 | 3 | 1b | 1a | IVa | 4 |
| 179821 | M | 73 | 3 | 0 | 0 | IIA | 12 |
| 179934 | M | 43 | 2 | 0 | 0 | I | 0 |
| 140313 | F | 56 | 2 | 1b | 0 | IIIA | 12 |
| 179793 | M | 57 | 3 | 0 | 0 | IIA | 2 |
| 180126 | M | 54 | 2 | 0 | 0 | I | 4 |
| 180026 | M | 54 | 4b | 0 | 0 | IIC | 9 |
| 180256 | M | 43 | 3 | 0 | 0 | IIA | 9 |
| 180070 | F | 35 | 2 | 0 | 0 | I | 6 |
| 180228 | F | 74 | 3 | 0 | 0 | IIA | 6 |
| 180267 | M | 45 | 3 | 0 | 0 | IIA | 2 |
| 180346 | F | 56 | 4a | 1b | 1c | IVC | 4 |
| 180301 | M | 71 | 3 | 0 | 0 | IIA | 9 |
| 180017 | F | 77 | 3 | 1b | 0 | IIIB | 4 |
| 180574 | F | 57 | 3 | 1a | 0 | IIIB | 4 |
| 180262 | M | 64 | 4b | 2a、1c | 1a | IVa | 9 |
| 180508 | F | 54 | 3 | 1a | 0 | IIIB | 3 |
| 172544 | M | 56 | 3 | 1a、1c | 1b | IVB | 4 |
| 180530 | F | 65 | 3 | 1a、1c | 1b | IVB | 9 |
| 173314 | M | 68 | 4b | 0 | 0 | IIC | 12 |
| 180345 | F | 76 | 3 | 1c | 1b | IVB | 9 |
| 180685 | F | 51 | 3 | 1b | 0 | IIIB | 6 |
| 148404 | F | 50 | 3 | 0 | 0 | IIA | 4 |
| 180853 | M | 51 | 1 | 0 | 0 | I | 9 |
| 180997 | M | 45 | 3 | 2a | 0 | IIIB | 4 |
| 180934 | M | 37 | 3 | 2a | 0 | IIIB | 6 |
| 180854 | M | 56 | 4a | 1a | 1a | IVa | 9 |
| 181032 | M | 43 | 2 | 0 | 0 | I | 6 |
| 176653 | M | 45 | 3 | 1b、1c | 1c | IVC | 4 |
| 182107 | M | 73 | 2 | 0 | 0 | I | 12 |
| 181343 | F | 77 | 2 | 0 | 0 | I | 8 |
| 181301 | M | 45 | 3 | 1a | 0 | IIIB | 12 |
| 181148 | M | 42 | 3 | 1c | 1a | IVa | 6 |
| 181430 | M | 57 | 4b | 0 | 0 | IIC | 9 |
| 181269 | M | 77 | 4a | 1b | 1b | IVB | 9 |
| 181532 | M | 69 | 2 | 0 | 0 | I | 9 |
| 181325 | M | 68 | 3 | 2b、1c | 0 | IIIC | 4 |
| 181582 | M | 54 | 3 | 0 | 0 | IIA | 2 |
| 181486 | F | 54 | 4a | 0 | 0 | IIb | 9 |
| 181392 | F | 52 | 3 | 1b | 1b | IVB | 6 |
| 181754 | M | 69 | 4b | 2a | 1b | IVB | 9 |
| 181701 | M | 66 | 3 | 0 | 0 | IIA | 0 |
| 181773 | F | 69 | 3 | 1b | 0 | IIIB | 6 |
| 181651 | M | 49 | 4a | 1a | 0 | IIIB | 9 |
| 181664 | F | 79 | T2 | 0 | 0 | I | 12 |
| 182145 | F | 73 | T4b | N2b | M1b | IVB | 4 |
| 181950 | M | 43 | T3 | 0 | M1a | IVA | 12 |
| 181782 | F | 90 | T3 | 0 | 0 | IIA | 4 |
| 182107 | M | 73 | T2 | 0 | 0 | I | 10 |
| 182018 | M | 51 | T2 | 0 | 0 | I | 4 |
| 181954 | M | 58 | T3 | 0 | 0 | IIA | 6 |
| 181642 | F | 81 | T2 | 0 | 0 | I | 4 |
| 181951 | F | 72 | T2 | 0 | 0 | I | 0 |
| 182130 | M | 70 | T3 | N1b | 0 | ⅢB | 3 |
| 182220 | M | 44 | T3 | 0 | 0 | IIA | 6 |
| 182172 | F | 65 | T4b | 0 | 0 | IIC | 9 |
| 182338 | M | 53 | T3 | N1b | 0 | ⅢB | 6 |
| 182408 | F | 62 | T3 | N2a | 0 | ⅢB | 1 |
| 182497 | F | 60 | T2 | 0 | 0 | I | 3 |
| 177135 | M | 50 | T3 | N1b | M1c | IVC | 6 |
