# Supplementary material for: The SOX9-MMS22L Axis Promotes Oxaliplatin Resistance in Colorectal Cancer
Source: Front Mol Biosci. 2021 May 27;8:646542. doi: 10.3389/fmolb.2021.646542 (PMC8191464; doi:10.3389/fmolb.2021.646542)
Supplement: Supplementary file 7 [file Table_2.docx]

**Supplementary table 2**

| **Name** | **Sources** | **Catalog number** | **Concentration** |
| --- | --- | --- | --- |
| SOX9 | ABclonal | A19710 | 1:1000 |
| OCT4 | CST | 2750S | 1:1000 |
| SOX2 | CST | 3579S | 1:1000 |
| GAPDH | CST | 5174S | 1:5000 |
| MMS22L | abcam | ab181047 | 1:1000 |
| NBS1 | CST | 14956S | 1:1000 |
| γH2AX | CST | 7631S | 1:1000 |
| Lamin B1 | CST | 13435S | 1:1000 |

**Primary antibody information**
